# Can Tanzanian Neurosurgeons Access Tanzanian Neurosurgical Literature? A Systematic Review and Survey of Neurosurgical Publications

**DOI:** 10.1227/neu.0000000000003570

**Published:** 2025-06-19

**Authors:** Romani R. Sabas, Julie Woodfield, Chibuikem Anthony Ikwuegbuenyi, Magalie Cadieux, Consolata Shayo, Zarina Shabhay, Happiness Rabiel, Beverly Cheserem, Joel Bwemelo, Drew N. Wright, Celestina S. Fivawo, Salome M. Maghembe, Kisitu Lawrence, Sengua Koipapi, Laurent Lemeri Mchome, Halinder S. Mangat, Roger Hartl, Hamisi Kimaro Shabani

**Affiliations:** * Department of Neurosurgery, Muhimbili Orthopedic Institute, Dar es Salaam, Tanzania;; ‡ Department of Neurological Surgery, Och Spine at New York Presbyterian Hospital, Weill Cornell Medicine, New York, New York, USA;; § Centre for Clinical Brain Sciences, University of Edinburgh, Edinburgh, UK;; ‖ Department of Neurosurgery, Washington University School of Medicine, St. Louis, Missouri, USA;; ¶ Department of Neurosurgery, Kilimanjaro Christian Medical Centre, Moshi, Tanzania;; # Neurosurgery Unit, Aga Khan University Hospital, Nairobi, Kenya;; ** Clinical Research, Training and Consultancy unit, Muhimbili Orthopedic Institute, Dar es Salaam, Tanzania;; †† Scholarly Communications Librarian Samuel J. Wood Library & C.V. Starr Biomedical Information Center, Weill Cornell Medical College, New York, New York, USA;; ‡‡ Muhimbili University of Health and Allied Science, Dar es Salaam, Tanzania;; §§ Department of Neurology, Division of Neurocritical Care, University of Kansas Medical Center, Kansas City, Missouri, USA

**Keywords:** Neurosurgery, Open access, Tanzania, Publishing

## Abstract

**BACKGROUND AND OBJECTIVES:**

Free and open access to research data and findings promotes equity in access to healthcare knowledge and equity in patient care and treatment. To benefit the health care of the population studied, research findings must be accessible to clinicians, academics, and policymakers serving those populations. The aim of this study was to assess the extent of published Tanzanian neurosurgical data and its accessibility to those practicing within the country.

**METHODS:**

A systematic review of all published neurosurgical studies from Tanzania was conducted. Authorship, funding, and open-access status were recorded. Tanzanian neurosurgeons were surveyed by telephone or in person about their methods of accessing literature.

**RESULTS:**

We identified 96 Tanzanian neurosurgical studies published in 42 journals between 1982 and 2023 with an exponentially increasing number of publications per year. Fifty-nine studies (62%) are available open access at the publisher. Open access publication is associated with Tanzanian first authorship (odds ratio = 2.6, 95% CI: 1.0-6.8) or last authorship (odds ratio = 2.7, 95% CI: 1.0-7.1). However, overall only 34 of 96 studies (35%) had Tanzanian first authors and 32 of 96 (33%) had Tanzanian last authors. We contacted 26 of 27 neurosurgeons working in Tanzania. None had in-country institutional library service access. One used a research initiative login to access neurosurgical literature, and 2 used institutional logins from outside Tanzania. Ten neurosurgeons (38%) reported alternative methods of accessing literature behind a paywall such as Sci-Hub or direct contact with authors. These methods could have given access to all but 9 of 96 neurosurgical studies (9%).

**CONCLUSION:**

Only 62% of Tanzanian neurosurgical literature is easily freely accessible to Tanzanian neurosurgeons, and 9% of all Tanzanian neurosurgical literature is extremely challenging to access for neurosurgeons working in Tanzania. Expanding open-access publishing, repositories, and publisher and institutional initiatives for equitable data and publication access are crucial for improving access to local data to improve patient care.

**ABBREVIATIONS:**
HICs: high-income countries
HINARI: Health Inter Network Access to Research Initiative
LMICs: low- or middle-income countries.

Free and open access to research data and findings promotes equity in access to healthcare knowledge and, consequently, equity in patient care and treatment. Recognizing the need for open access to scientific data, many public research funders came together in Plan S in 2021 to mandate that scientific research funded by public or private grants from national or international funding bodies, and research councils must be made available through open-access platforms, journals, or repositories.^1^

However, partnerships between neurosurgical departments in low- or middle-income countries (LMICs) and high-income countries (HICs) may be funded philanthropically by private or charity donors. These partnerships can enable neurosurgical knowledge transfer, skills learning, and capacity strengthening through clinical and research activities.^2-5^ Although partnerships can make substantial contributions to global neurosurgical knowledge, for maximum impact in LMICs, research findings from these collaborations need to be available to researchers, clinicians, and policy makers in LMICs. Difficulties in making research findings from LMIC-HIC partnerships freely available may arise through lack of established open-access policies in the partnerships, lack of access to LMIC schemes because of the research being performed with HIC partners,^6,7^ lack of recognition of the importance of open-access publishing, or prohibitive open-access publisher costs not budgeted for by either partner.^8-10^

Various initiatives aiming to make research open access for developing countries exist. One example is the Health Inter Network Access to Research Initiative (HINARI), established in 2001, and part of Research4Life.^11^ This provides institutions from eligible countries with access to participating journals and databases.^12,13^ However, it remains uncertain whether those planning and delivering health care are aware of or have easy access to these opportunities. Given the high volume of patients with brain and spine trauma, hydrocephalus, spina bifida, and other neurosurgical conditions in Tanzania, the rapidly expanding neurosurgical workforce in Tanzania, and the need for preventative and public health care, sharing knowledge through access to the findings of studies performed within Tanzania is crucial for streamlining the development of consistent, high-quality patient care in the region.^14^

The authors of this study have been involved in a long-term neurosurgical collaboration between Muhimbili Orthopedic Institute (in a LMIC) and Weill Cornell Medicine (in a HIC) for over 10 years.^15,16^ The aim of this study was to assess whether published Tanzanian neurosurgical studies are freely available to those working in neurosurgery in Tanzania and whether the source of funding or involvement of international collaboration is associated with publishing practices. We also aimed to assess how Tanzanian neurosurgeons access published data about the population being treated.

## METHODS

### Systematic Review

The systematic review followed the Preferred Reporting Items for Systematic Review and Meta-Analysis guidelines.^17^ It was registered on the Prospero Systematic Review Database on September 4, 2023.

### Eligibility Criteria

#### Inclusion Criteria

We included all published studies concerning neurosurgical conditions in Tanzania. Neurosurgical conditions included a range of disorders typically managed by neurosurgeons, such as hydrocephalus, traumatic brain injury, dysraphism, spine trauma, neurovascular conditions, and neuro-oncology. We included studies published in any language without requiring the journal to be indexed in PubMed or any other database. All study types, eg, primary data articles, descriptions of operative procedures or equipment use, reviews, editorials, commentaries, protocols, and case reports, were included. There were no date restrictions. Studies had to include or analyze data or findings from Tanzania.

#### Exclusion Criteria

We excluded studies that focused on neurological conditions such as epilepsy or stroke where neurosurgical treatment was not analyzed or considered, research authored by Tanzanians studying neurosurgical care in other countries, and studies in which Tanzanian data contributed <50% of the data set.

### Search Strategy

A search was conducted across 6 databases: PubMed, Web of Science, Embase, Scopus, MEDLINE, and the WHO database. Additional searches were conducted in African Journals Online and Google Scholar. The search strategy was designed to identify all Tanzanian neurosurgical literature, regardless of journal indexing, and included names of all current and previous known Tanzanian neurosurgeons. The search strategy for PubMed is detailed in Table 1. Search strategies for the other databases are provided in the **Supplemental Digital Content 1** (http://links.lww.com/NEU/E830). The reference lists of all included studies were screened to identify any further relevant studies. The final search was completed for each database on October 06, 2023.

**TABLE 1.** The Search Strategy for PubMed Database

((“Neurosurgical Procedures”[Mesh] OR “Neurosurgery”[Mesh] OR neurosurg*) AND (“Tanzania”[Mesh] OR tanzan*)) OR (“Kaale Aingaya”[au] OR “Kinghomella Alpha”[au] OR “Miranda Alvin”[au] OR “Kivevele Boniface”[au] OR “Bureta Costansia”[au] OR “Mayaya Gerald”[au] OR “Hamis Kinyerero”[au] OR “Hamisi Shabani”[au] OR “Rabiel Happiness”[au] OR “Humba Henry”[au] OR “Lubuulwa James”[au] OR “Ngerageza Japhet”[au] OR “Mtei John”[au] OR “Mzimbiri Juma”[au] OR “Mchome Laurent”[au] OR “Ndossi Maxigama”[au] OR “Mazoko Mugisha”[au] OR “Rutabasibwa Nicephorous”[au] OR “Makundi Raymond”[au] OR “Majige Rebecca”[au] OR “Ahmada Saidi”[au] OR “Morales Vladimir”[au] OR “Kiloloma Wanin”[au] OR “Shabhay Zarina”[au])

### Study Selection

The search results were transferred to the Covidence™ online systematic review platform (access facilitated through Weill Cornell Medicine), and duplicates were removed. Abstracts and titles were independently screened by 2 researchers, and full texts of studies were reviewed independently by 2 researchers against predefined eligibility criteria. Any discrepancies were resolved through discussion within the team.

### Data Extraction

Two researchers independently extracted study data. Discrepancies were reviewed and resolved by involving a third researcher. Extracted details included first, second, and last authors' names; study titles; countries of affiliation; journal names; publication years; and funding sources listed. We recorded these in an Excel spreadsheet. The authors' first listed affiliation was used as their nationality as affiliation would more likely determine funding availability. Funding sources were identified only if specified in the published article.

### Data Analysis

Calculated data points included author gender, the journal's impact factor, whether the journal was indexed in Medline, the subspecialty topic of the article, type of study, any involvement of foreign authors or institutions, World Bank classification of the country of affiliation, and open-access status. Sex was determined through knowledge of the author or an internet search for the few authors not known to the authors of this review. Journal impact factors for the year 2023 were determined using the Clarivate Science Citation Index.^18^ Topic was assigned to neurosurgical subspecialty categories of the following: hydrocephalus; spine trauma; traumatic brain injury; neuro-oncology; spine (nontraumatic); neurovascular, infectious, neurosurgical education; and other. Study type was recorded as follows: primary data article (eg, cohort study, randomized or nonrandomized trial, case series, and case report), review or commentary, and protocol. A foreign author or institution was identified as involved in the study if there were any authors in any position who were not affiliated with a Tanzanian institution. New World Bank countries classification data of 2022 were used to determine the economies of the countries the authors were affiliated with.^19^ The open-access status of the articles was assessed by 2 independent researchers who did not have any institutional logins and searched online from Tanzania. If the article was freely accessible at the publisher, this was recorded. We used Unpaywall and online searching to check for any final author-accepted articles available in repositories.^13^ Unpaywall searches using DOI for open-access publications across institutional repositories and databases.^13,20^ One Tanzanian author used their HINARI login to assess which articles were available in Tanzania using Tanzanian HINARI access. As several Tanzanian neurosurgeons reported using Sci-Hub, we also searched for all articles in Sci-Hub from Tanzania and recorded which were available.

### Survey Methods

All currently employed Tanzanian neurosurgeons were contacted in person or by telephone. Survey questions are in the **Supplemental Digital Content 1** (http://links.lww.com/NEU/E830). Data were gathered about sources or platforms used for searching or accessing neurosurgical studies and any affiliations or logins that facilitate access to articles behind paywalls. We also asked about the number of articles authored and the availability of open-access options for publication for those studies.

### Statistical Analysis

Descriptive analysis was conducted using SPSS Version 23. Numbers and proportions were reported. To compare categorical data, odds ratios (OR) and 95% CIs were calculated, along with the χ^2^ test and Fisher exact test where needed. Statistical significance was defined as a *P*-value of <.05.

### Data Availability Statement

All data related to the analysis of this work is available upon request from the corresponding author.

## RESULTS

### Description of Studies

**FIGURE 1.**
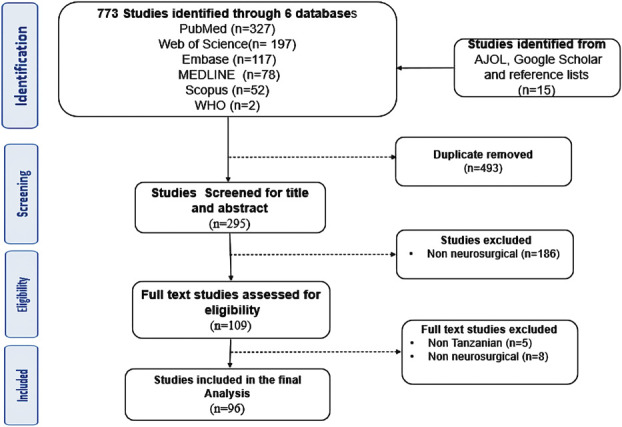
Preferred Reporting Items for Systematic Review and Meta-Analysis flow diagram of studies. AJOL, African Journal Online; WHO, World Health Organization.

**FIGURE 2.**
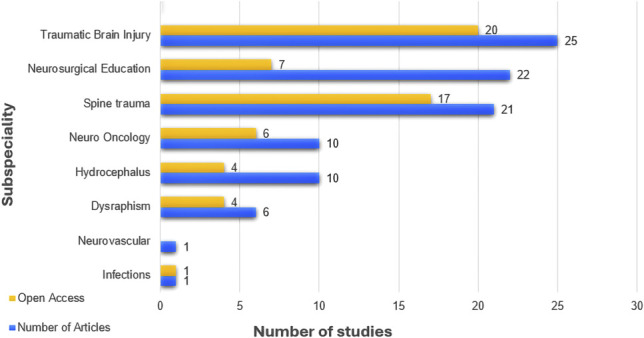
Open-access publishing by research topics. The blue bars indicate the number of articles published for each topic. The yellow bars represent the number of open-access articles for each topic. Articles categorized as “Neurosurgical education” include those assessing teaching and training challenges and capacity-strengthening initiatives.

In total, 788 studies were identified by the search, with 96 meeting the eligibility criteria for inclusion in the final analysis (see PRISMA Flow Diagram in Figure 1). The most frequently investigated topic was traumatic brain injury (25/96, 26 %). This was followed by neurosurgical education (including capacity strengthening, research, or educational initiatives, 22/96, 23%), and traumatic spine injury (21/96, 22%). Other neurosurgical subspecialties were less commonly studied, as shown in Figure 2 and Table 2. The primary data studies were cohort studies (67/96, 70%), and case series or reports (20/96, 21%, Table 2). No comparative trials were identified.

**TABLE 2.** Description of Published Tanzanian Neurosurgical Literature

	Total (%)	Open access (%)	Odds ratio (95% CI)	*P*-value
	96 (100)	59 (61.5)		
Foreign institutions involved				
Yes	74 (77.1)	42 (56.8)	0.4 (0.13-1.16)	.09
No	22 (24.0)	17 (77.3)		
Funding source listed				
Yes	15 (16.3)	10 (66.7)	1.3 (0.41-4.20)	.60
No	81 (84.3)	49 (60.4)		
Affiliations of authors				
First author				
Tanzanian	34 (35.4)	26 (76.5)	2.6 (1.03-6.80)	.04
Non-Tanzanian	62 (64.5)	33 (53.2)		
Second author				
Tanzanian	44 (45.8)	30 (68.1)	1.7 (0.75-4.10)	.20
Non-Tanzanian	52 (54.2)	29 (55.8)		
Last author				
Tanzanian	32 (33.3)	24 (75.0)	2.7 (1.00-7.10)	.05
Non-Tanzanian	64 (66.7)	35 (54.7)		
Gender of authors				
First author				
Male	78 (81.3)	45 (57.7)	0.4 (0.13-1.51)	.19
Female	18 (18.7)	14 (77.8)		
Second author				
Male	73 (76.0)	41 (56.2)	0.3 (0.11-1.03)	.06
Female	23 (24.0)	18 (73.3)		
Last author				
Male	73 (76.0)	45 (61.6)	0.8 (0.28-2.10)	.70
Female	23 (24.0)	14 (60.8)		
Journal indexed in PubMed				
Yes	88 (91.7)	55 (62.5)	1.7 (0.39-7.12)	.49
No	8 (8.3)	4 (50.0)		
Journal of publication				
Word Neurosurgery	26 (27.1)	6 (23.1)		
Neurosurgical Focus	5 (5.2)	5 (100.0)		
Journal of Neurosurgery	5 (5.2)	2 (40.0)		
Tanzania Medical Journal^a^	3 (3.1)	0 (0.0)		
Global Spine Journal	2 (2.1)	2 (100.0)		
PLoS One	2 (2.1)	2 (100.0)		
Lancet	1 (1.0)	0 (0.0)		
Others^b^	52 (54.2)	37 (71.2)		
Subspecialty				
Traumatic brain injury	25 (26.0)	20 (80.0)		
Neurosurgical education	22 (22.9)	7 (31.8)		
Traumatic spine injury	21 (21.8)	17 (81.0)		
Neuro-oncology	10 (10.4)	6 (60.0)		
Hydrocephalus	10 (10.4)	4 (40.0)		
Dysraphism	6 (6.2)	4 (66.7)		
Infections	1 (1.0)	1 (100.0)		
Neurovascular	1 (1.0)	0 (0.00)		
Article study type				
Cohort	67 (69.8)	43 (64.2)		
Case report or series	20 (20.8)	14 (70.0)		
Review or commentary	9 (9.4)	2 (22.2)		

a The journal states it is fully open access but payment was required to view the articles.

b Journal of Neurosurgery Pediatric, International Journal of Surgery Case Reports, Brain and Spine Journal, Tanzania Journal of Health Research, Journal of Neurotrauma, East Africa Medical Journal, African Health Sciences, Journal of Neurosurgery Spine, World Journal of Pediatric Surgery, Telemedicine Journal of E Health, Spinal Cord, Springer Nature, Pan African Medical Journal, Neurosurgery, Neurocirugia, Mega Journal Oncology, Journal of Medical Case Reports, International Journal of Spine Surgery, International Journal of Injury Control and Safety Promotion, Egyptian Journal of Neurosurgery, East and Central African Journal of Surgery, East African Health Research Journal, Disability and Rehabilitation, Disability & Society, Cureus, Case Report in Womens Health Journal, BMJ Case Series, BMC Neurology, East and Central African Journal of Surgery, American Journal of Clinical Pathology, American Journal of Medical Genetics and African Journal of Emergency Medicine.

### Authorship

**FIGURE 3.**
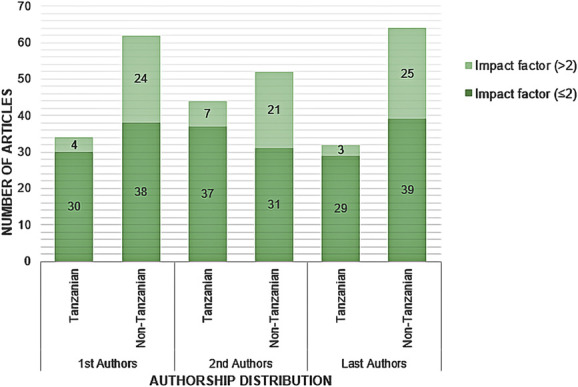
Authorship of Tanzanian neurosurgical studies. First, second and last authors and whether their primary affiliation is a Tanzanian institution. Light green parts of the bar represent studies published in a journal with an impact factor of ≤2, and deep green parts of the bar represent studies published in a journal with an impact factor of more than 2.

A lower proportion of authors were Tanzanian than non-Tanzanian. Tanzanians were first authors in 34 of 96 studies (35%), second authors in 44 of 96 studies (46%), and last authors in 32 of 96 studies (33%) (Figure 3). There was a high proportion of male authorship (first author: 78/96, 81%; second author: 73/96, 76%; last author: 73/96, 76%, Table 2)

### Publication

**FIGURE 4.**
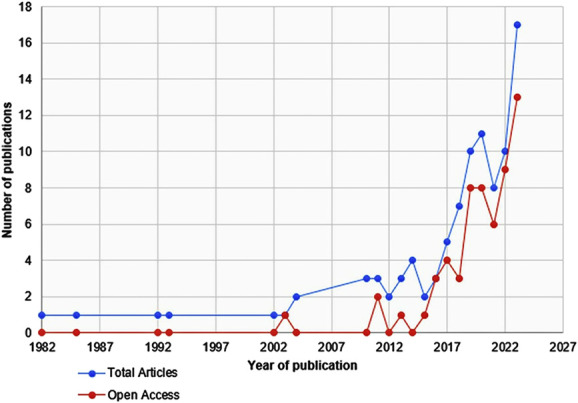
Open-access publishing over time. The blue line represents the overall number of neurosurgical articles published each year, whereas the red line represents the number of open-access articles published each year.

Studies were published in 42 journals between 1982 and 2023. There was an exponential increase in the number of Tanzanian neurosurgical studies published each year from 2002 (Figure 4). The journal publishing by far the most Tanzanian neurosurgical literature was World Neurosurgery, accounting for 26 of 96 (27%) (Table 2). Ninety percent (38/42) of the journals publishing Tanzanian neurosurgical literature are indexed in PubMed. All journals where studies were published undertook peer review before publishing. The median impact factor of journals publishing Tanzanian neurosurgical literature was 2. First, second, or last authors affiliated with Tanzanian institutions were less likely to publish in a journal with impact factor of more than 2 compared with those affiliated with non-Tanzanian institutions (first authors Tanzanian: 12% vs 39% Non-Tanzanian, OR = 0.21, 95% CI: 0.66-0.67, *P* = .01; second authors Tanzanian: 16% vs 40% Non-Tanzanian, OR = 0.28, 95% CI: 0.11-0.74, *P* = .01; last authors Tanzanian: 9% vs 39% Non-Tanzanian, OR = 0.16, 95% CI: 0.04-0.59, *P* = .006, as illustrated in Figure 3).

### Open-Access Status

**FIGURE 5.**
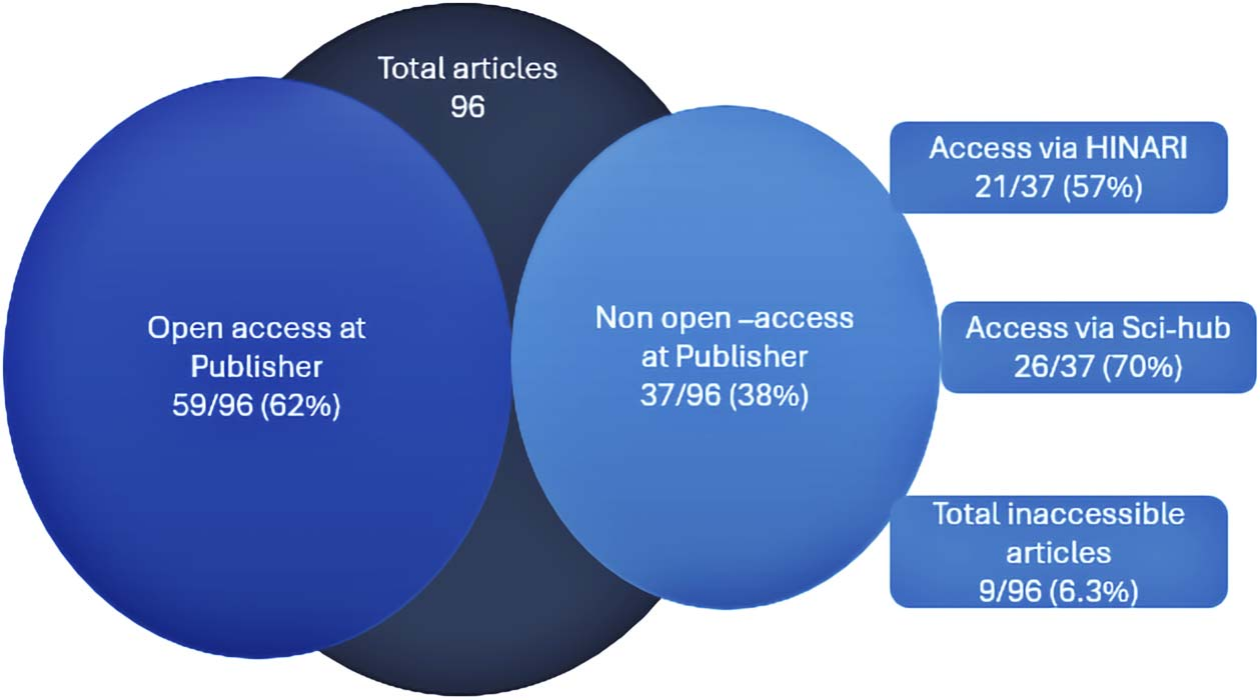
Open-access status of Tanzanian Neurosurgical Literature and Alternative Access methods. HINARI, Health Inter Network Access to Research Initiative.

Of the 96 articles, 59 (62%) were available for open access at the publisher or journal website. There was a gradual increase over time in studies published open access along with the total number of studies (Figure 4). For those not freely available online (non–open access, n = 37), 21 (56%) could be accessed through the HINARI Research4Life platform, and 26 (70%) were available through Sci-Hub from within Tanzania. In total, only 9 of 96 (9%) were found to be inaccessible to any method of literature access in Tanzania without paying a fee per article (Figure 5). Online open-access availability at the publisher was higher for articles with first and last authors from Tanzania (Tanzanian first author 26/34, 76.5% vs 33/62 53.2%, OR = 2.6, 95% CI: 1.03-6.80, *P* = .04, Tanzanian last author: 24/32, 75% vs 35/64, 55%, OR = 2.7, 95% CI: 1.00-7.10, *P* = .05). The difference in open-access availability between whether all authors were from Tanzania (17/22, 77%) or foreign authors were involved (42/74, 57%, OR = 0.4, 95% CI: 0.13-1.16, *P* = .09, Table 2) was not statistically different. There was no difference in open-access status when a funding source was stated in the publication (funding source stated: 10/15, 67% vs no funding source stated 49/81, 60% OR = 1.3, 95% CI: 0.41-4.20, *P* = .60).Funding sources were listed in a total of 15 of 96 studies (16%).

### Access to Tanzanian Studies

Tanzania has 27 practicing neurosurgeons, with 82% (22/27) being male. Most neurosurgeons are located in Dar es Salaam (17/27, 63%), with 13 of 27 (48%) at Muhimbili Orthopedic Institute. 26 of 27 Tanzanian neurosurgeons (96%) were successfully contacted. To search for Tanzanian neurosurgical studies, most neurosurgeons reported using Google (23/26, 88%), but PubMed (11/26, 42%) and Google Scholar (12/26, 46%) were also mentioned. Only 3 of 26 neurosurgeons (12%) had access to an institutional login. This was HINARI in 1 case and an institutional login for an institution outside Tanzania in 2 cases. No neurosurgeon reported having a Tanzanian institutional login to access medical or neurosurgical literature. When presented with articles behind a paywall, 13 of 26 neurosurgeons (50%) reported only reading the abstracts. 10 of 26 neurosurgeons (38%) used Sci-Hub and 3 of 26 (12%) asked friends or colleagues with institutional logins to send them the articles or asked those who had been involved in the projects to send them the articles. Fifty percent (13/26) of Tanzanian neurosurgeons reported publishing research articles on Tanzanian neurosurgery, and of these, 85% (11/13) reported publishing at least 1 article open access. Sources for open-access publication fees reported were journal waivers for LMICs (2/11, 18%), colleagues (5/11, 45%), and institutions abroad (4/11, 36%).

## DISCUSSION

Tanzanian neurosurgical literature continues to grow year on year (Figure 4), increasing the availability of local data and expertise on which to make neurosurgical clinical and policy decisions. The high proportion of studies investigating traumatic brain and spine injuries likely reflects both the high incidence of these injuries in Tanzania^21-24^ and the historical development of neurosurgical services around trauma care.^21^ For study findings to impact on health care, the results need to be accessible. Overall, 59 of 96 (62%) Tanzanian neurosurgical published studies were open-access online at the publisher.

The proportion of Tanzanian neurosurgical studies available open access is higher than the 28% reported by a large-scale analysis of all published scholarly articles across all subjects globally.^13^ Articles authored by low-income countries,^25^ particularly from sub-Saharan Africa, have been identified as being more likely to be published as open access than those from HICs.^25^ We found that articles with Tanzanian first or last authors were more likely to be published as open access than those authored by non-Tanzanians. Articles with Tanzanian authorship may be eligible for open-access publication fee waivers for authors from LMICs if no HIC authors are involved,^12^ but those working in Tanzania may also be more aware of the importance of open-access publication for dissemination of study results because of every day struggles accessing literature compared with foreign collaborators whose home institutions may provide seamless single login library access to multiple publishers.

Only one Tanzanian neurosurgeon reported accessing studies behind a paywall using HINARI and 2 used foreign institutional logins. Research4Life provides access to biomedical literature from many international publishers through HINARI.^11,26^ With a HINARI login and open access, 70 of 96 studies (73%) would have been available. However, institutional awareness and library and email support to manage logins are needed for HINARI access, and the national neurosurgical referral center where half of Tanzanian neurosurgeons practice was not registered at the time of this study. Even with lowered or no subscription fees, the need for institutional library support or infrastructure such as institutional email addresses to participate in existing schemes can hinder literature access.^26-28^ As HINARI access is restricted to not-for-profit institutions, neurosurgeons working in private practice also might not benefit from this initiative.^29^ More neurosurgeons (10/26, 38%) resourcefully used their contacts or services such as Sci-Hub^28,30^ to access the data they need to plan and inform patient care. With Sci-Hub, 85 of 96 studies (89%) were available within Tanzania, reflecting the controversial reality of every day choices to access scientific data. We did not ask about contacting authors for full-text articles through services such as Research Gate,^31^ and none of our respondents mentioned this as a way of acquiring full-text articles but many reported using informal networks of colleagues or friends for access. None of the neurosurgeons reported paying at journal or publisher websites for access to single articles, and we did not explore attitudes or barriers to this, but 13 of 26 neurosurgeons (50%) reported only reading the abstract when they met a paywall.

We found a low rate of Tanzanian first (35%) and last authorship (33%) for Tanzanian neurosurgical studies, consistent with findings from other studies of neurosurgical research output from LMICs.^12,32,33^ It has been suggested that this arises from more institutional and employer focus on clinical and technical skills above research skills in LMICs, lack of time, opportunities, or funding for learning research skills and carrying out research studies, language barriers, under-representation on editorial boards or as reviewers, and power dynamics within international collaborations.^3,12,28,32-34^ Although many studies have identified that international collaboration is associated with a higher rate of open-access publishing,^25^ this was not the case in our study.

Many international or national public funding bodies mandate immediate open-access funding through initiatives such as Plan S, which was supported by the European Research Council,^1,27^ and many global health initiatives would expect open-access publication. We found only 10 of 96 Tanzanian neurosurgical studies (10%) declared public funding, mostly from the US National Institute of Health, and declaration of funding was not associated with open-access publishing. Requirements and collaboration between funders, publishers, and institutions have improved speed of open-access publication, as well as platforms, repositories, and journals providing open-access funding. Although the National Institute for Medical Research in Tanzania promotes sharing of findings with participants and review of published articles of approved studies before publication, in line with most other Institutional Review Boards open-access publishing is not mandated by ethical approval.^35^ We found that even in the most recent year studied, only 13 of 17 Tanzanian neurosurgical studies (76%) were available open access, which is not much higher than the overall proportion across all years, suggesting that much still needs to be performed to improve this.

### Limitations

This study is limited by the data collection methods. Although we included a wide range of database sources to identify studies published in journals that may not be indexed in PubMed or other major databases, we may still have missed some neurosurgical articles or data sets published. We used the first affiliation listed as the author's nationality because we thought this most likely represented their primary place of work and therefore the resources available to them. However, many authors had affiliations across different countries, and many doctors work and live in different countries. This could affect our assessment of Tanzanian authorship.

## CONCLUSION

Access to local data is crucial for improving individual, institutional, and national patient care and for national and international policymaking. The United Nations' Sustainable Development Goals emphasize the importance of gaining access to and actively participating in creating and sharing knowledge as critical drivers for economic, social, and environmental development.^36^ However, approximately one-third of Tanzanian neurosurgical literature is behind a paywall that half of Tanzanian neurosurgeons struggle to access. We suggest expanding institutional, funder, publisher, and collaboration support of initiatives such as Plan S, HINARI, and open-access fee waivers to improve this.

## SUPPLEMENTAL DIGITAL CONTENT

**Supplemental Digital Content 1.** **Database search strategies:** Search strategies for each database searched in the systematic literature review. **Survey questions:** Can Tanzanian Neurosurgeons access Tanzanian Neurosurgical Literature? Survey administered to Tanzanian neurosurgeons.
